# Multi-tissue transcriptomic characterization of endogenous retrovirus-derived transcripts in *Capra hircus*

**DOI:** 10.3389/fgene.2025.1544330

**Published:** 2025-03-19

**Authors:** Ming-Di Li, Hu-Rong Li, Shao-Hui Ye

**Affiliations:** ^1^ Department of Animal Breeding and Reproduction, College of Animal Science and Technology, Yunnan Agricultural University, Kunming, China; ^2^ Kunming Institute of Zoology, Chinese Academy of Sciences, Kunming, China

**Keywords:** transposable element, endogenous retrovirus, *Capra hircus*, goat, transcriptome

## Abstract

**Background:**

Transposable elements (TEs, or transposons) are repetitive genomic sequences, accounting for half of a mammal genome. Most TEs are transcriptionally silenced, whereas some TEs, especially endogenous retroviruses (ERVs, long terminal repeat retrotransposons), are physiologically expressed in certain conditions. However, the expression pattern of TEs in those less studied species, like goat (*Capra hircus*), remains unclear. To obtain an overview of the genomic and transcriptomic features of TEs and ERVs in goat, an important farm species, we herein analyzed transcriptomes of ten *C. hircus* tissues and cells under various physiological and pathological conditions.

**Method:**

Distribution of classes, families, and subfamilies of TEs in the *C. hircus* genome were systematically annotated. The expression patterns of TE-derived transcripts in multiple tissues were investigated at subfamily and location levels. Differential expression of ERV-derived reads was measured under various physiological and pathological conditions, such as embryo development and virus infection challenges. Co-expression between ERV-reads and their proximal genes was also explored to decipher the expression regulation of ERV-derived transcripts.

**Results:**

There are around 800 TE subfamilies in the goat genome, accounting for 49.1% of the goat genome sequence. TE-derived reads account for 10% of the transcriptome and their abundance are comparable in various goat tissues, while expression of ERVs are variable among tissues. We further characterized expression pattern of ERV reads in various tissues. Differential expression analysis showed that ERVs are highly active in 16-cell embryos, when the genome of the zygote begins to transcribe its own genes. We also recognized numerous activated ERV reads in response to RNA virus infection in lung, spleen, caecum, and immune cells. CapAeg_1.233:ERVK in chromosome 1 and 17 are dysregulated under endometrium development and infection conditions. They showed strong co-expression with their proximal gene *OAS1* and *TMPRSS2*, indicating the impact of activated proximal gene expression on nearby ERVs.

**Conclusion:**

We generated ERV transcriptomes across goat tissues, and identified ERVs activated in response to different physiological and pathological conditions.

## 1 Introduction

Transposable elements (TEs, also called transposons) are mobile genetic elements consist of repetitive sequences, accounting for about half of a mammal genome (Dong et al., 2013; de Koning et al., 2011). According to the origin and mobile type, TEs could be classified into several classes and families, including DNA transposons, long interspersed nuclear elements (LINEs), short interspersed nuclear elements (SINEs), and the long terminal repeat (LTR) family, which mainly contains endogenous retroviruses (ERVs) (Lanciano and Cristofari, 2020). TEs play significant roles in shaping the size, structure and function of mammal genome. It is well recognized that the host could leverage TEs to facilitate specific biological processes. For instance, the ERV-derived envelope protein, syncytia, induces the fusion of placental trophoblast cells (Chuong, 2018), driving the evolution of placental mammals (Mi et al., 2000). Additionally, a large proportion of TEs, though being non-coding sequences, act as enhancers in host genome to regulate the expression of coding genes in various processes.

At the transcriptome level, most TEs, especially ERVs which have the potential to code for proteins, are silenced and located in heterochromatin region in the genome. When not properly silenced, they may be activated (transcribed) under certain conditions. Such abnormal TE expression may contribute to the pathogenesis of various diseases (Horvath et al., 2017). Although TEs are non-negligible in genomic analysis, they are typically ignored in transcriptomic analysis. Since TE-derived reads are highly repetitive, rendering complexities and uncertainty in attributing ambiguously aligned short reads to the exact elements, TE-associated reads are often discarded in RNA sequencing data analyses.

Because of the above technical challenges, TE-derived reads are somewhat overlooked in typical genomic and transcriptomic analyses, especially in less studied species. In the genome of *Capra hircus* (goat), the distribution and characterization of TEs are not well annotated. Whether, when, and how those TEs are expressed in goat tissues are also unclear. To gain an overview of the genomic and transcriptomic features of TEs in *C. hircus*, we herein analyzed TE- and ERV- derived transcripts at both subfamily and location levels, in a dozen bulk RNA-seq datasets of ten *C. hircus* tissues and cells under various physiological and pathological conditions. Since TEs, especially ERVs, are physiologically expressed in embryos and placenta, we initially analyzed whether any ERVs were differentially expressed during embryo development, as well as in endometrium, where the expression regulation might be regulated cooperatively. We then checked infection related datasets to investigate whether ERVs were dysregulated in response to infection, as external stress might be a source for endogenous TE activation. We generated detailed annotation files for genomic and transcriptomic analyses for goat genome, and assessed the genome-wide expression patterns of ERVs across goat tissues in various conditions, providing a reference ERV atlas for TE research in goats.

## 2 Methods

### 2.1 Study design

**FIGURE 1:**
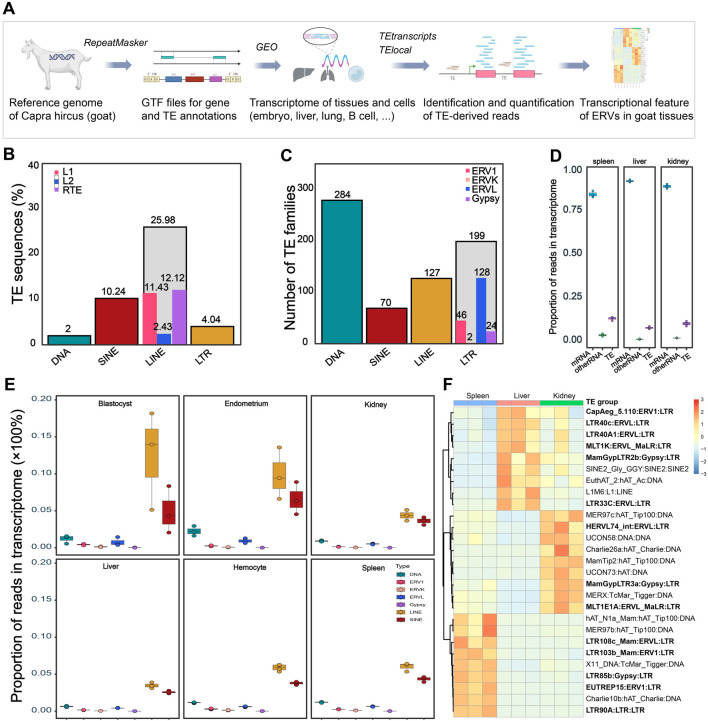
Genomic and transcriptomic features of *Capra hircus* TEs. **(A)** Workflow of the current study. The diagram was partially created using the web-based tool BioRender. GTF, Gene Transfer Format; TE, transposable elements; GEO, Gene Expression Omnibus; ERV, endogenous retrovirus. **(B)** Percent of TE sequences (%) in the *Capra hircus* genome. Source data for the ratio of TE subfamilies was shown in Supplementary Table S1. **(C)** Number of each type of TE subfamilies in the transcriptome of different tissues and cells. Average number of each type of TEs in transcriptomes across tissues were presented. Source data was shown in Supplementary Table S2. **(D)** Ratio of each type of TEs in total transcriptome of different tissues. Plots in blue refer to mRNA, plots in purple refer to TE-derived RNA, and plots in green refer to other types of RNA. Source data was retrieved from GSE93855. **(E)** Boxplots showing the abundance of TE-derived reads count in the transcriptome of several goat tissues: blastocyst (GSE129742), endometrium (GSE184110), kidney (GSE93855), liver (GSE93855), spleen (GSE93855), and hemocyte (GSE132429). **(F)** Heatmap of tissue specific TEs in kidney, liver, and spleen from GSE93855; log2 transformed counts were used for expression quantification; color scale bar showing the range of normalized expression of each TE. Tissue-specific of LTRs (ERVs) were marked in bold.

We initially obtained the Ensembl curated *C. hircus* genome assembly ARS1 (GCA_001704415.1), which was well annotated for genomic features and was widely-used, and then annotated the TEs using RepeatMasker (http://www.repeatmasker.org). The annotated GTF (Gene Transfer Format) file was then used for subsequent TE identification in the transcriptional analyses. RNA sequencing of goat tissues was comprehensively explored in the Gene Expression Omnibus (GEO, https://www.ncbi.nlm.nih.gov/geo/browse/), resulting in 12 datasets from 10 *C. hircus* tissues and cells under various physiological and pathological conditions. Raw sequencing reads were processed using TEtranscripts (Jin et al., 2015) and TElocal (https://github.com/mhammell-laboratory/TElocal). Genomic and transcriptomic features, including TE types, genomic distributions, and expression patterns, were investigated across goat tissues (Figure 1A).

### 2.2 Data sources

The RNA-seq datasets utilized in this study were sourced from the GEO repository (accession numbers were listed in Table 1). In brief, GSE69812 includes 6 fetal skin samples from normal and hyperpigmented goats (Ren et al., 2016), while GSE164100 (Bhat et al., 2021) includes 19 skin biopsies containing secondary hair follicles from ten 24-month-old male Pashmina goats, repeatedly sampled at resting phase (telogen) and active growth phase (anagen). GSE93855 analyzed kidney, liver, and spleen of three unrelated adult female goats at high- and low-altitude (Tang et al., 2017). Since ERVs are physiologically expressed in the placenta, we analyzed expression pattern of ERVs in embryos (GSE129742) (Li et al., 2020), as well as in ovary (GSE120144) (Liu et al., 2018) and endometrium (GSE108557 and GSE184110) (Liu et al., 2021), where the gene expression might be regulated cooperatively. In addition to these tissues under physiological conditions, we also investigated infection related datasets. GSE130552 includes 12 samples from lung, spleen, and caecum of control and Peste-des-petits-ruminants virus (PPRV) infected goats at 9 days-post-infection, whereas GSE132429 analyzed monocytes and lymphocytes from PPRV infected goats (Wani et al., 2019). GSE121725 includes expression profiling of skin fibroblast cells in response to ORF virus infection, and GSE30379 analyzed mammary epithelial cells in response to *Mycoplasma agalactiae* challenge, and GSE117799 analyzed peripheral blood mononuclear cells from goats infected with *Mycobacterium avium subsp. Paratuberculosis* (Berry et al., 2018). To further investigate the distribution and regulation of TEs in goat, assays for transposase-accessible chromatin using sequencing (ATAC-seq) in goat tissues and cells were also screened in literature and databases. One reference ATAC-seq data from goat liver and CD4^+^ and CD8^+^ T cells, generated by the Functional Annotation of Animal Genomes (FAANG) project was obtained. Processed summary data, with quantified ATAC-seq peaks and chromosomal coordinates of the open regions, were downloaded from the original reference (Foissac et al., 2019), to detect TEs distributions in the context of chromatin landscape in goat tissues and cells.

**TABLE 1:** Characteristics of analyzed RNA-seq datasets from various goat tissues.

Condition	Tissue/cell	Sample description	Sample size	Mapping rate	Data source	SRA accession
Physiological conditions	Skin	Fetal skin sampled from the normal and hyperpigmentated goats	6	75.70%	GSE69812	SRP059421
		Skin biopsies containing secondary hair follicles from 24-months old male Pashmina goats, repeatedly sampled at resting phase (telogen) and active growth phase (anagen)	19	77.7%	GSE164100	SRP299900
	Liver, spleen, kidney	kidney, liver, and spleen of three unrelated adult female goats at high- and low- altitude	12	89.03%	GSE93855	SRP055702
	Oocyte and embryo	Matured oocytes and *in vivo* developed embryos from the 2-cell to the blastocyst stages	21	76.80%	GSE129742	SRP192394
	Ovary	Ovary at follicular and luteal phases	10	85.70%	GSE120144	SRP163266
	Endometrial epithelium	Peri-implantation endometrial epithelium at 6-day,16-day, and19-day of pregnancy	9	75.70%	GSE108557	SRP127612
	Endometrial epithelial cell	Endometrial epithelial cells with or without IFNT treatment	6	85.70%	GSE184110	SRP337048
Pathological conditions	Lung, spleen and caecum	Lung, spleen, and caecum of control and Peste-des-petits-ruminants virus (PPRV) infected goats at 9 days post infection	12	77.10%	GSE130552	SRP194369
	Monocytes and lymphocytes	Monocytes and lymphocytes from Peste-des-petits-ruminants virus (PPRV) infected goats	16	75.20%	GSE132429	SRP200956
	Skin fibroblast cells	Skin fibroblast cells in response to Orf virus infection	6	79.50%	GSE121725	SRP166752
	Mammary epithelial cells	Mammary epithelial cells to *Mycoplasma* agalactiae challenge at different time-points (3 h, 12 h, and 24 h) post infection	12	80.30%	GSE30379	SRP007396
	Peripheral blood mononuclear cells	PBMC from goats that were vaccinated and infected with *Mycobacterium avium* subsp. Paratuberculosis	15	86.30%	GSE117799	SRP155536

Note, raw RNA-seq data retrieved from Gene Expression Omnibus (GEO, https://www.ncbi.nlm.nih.gov/geo/browse/).

### 2.3 Genomic annotation of TEs

Genomic annotation of TEs was conducted according to literature (Lanciano and Cristofari, 2020; Tarailo-Graovac and Chen, 2009; Dong et al., 2015; Bourque et al., 2018). The Ensembl curated assembly ARS1 was used for genome mapping and annotation. The reference sequence (FASTA file) was accessed at https://ftp.ensembl.org/pub/release-112/fasta/capra_hircus/dna/Capra_hircus.ARS1.dna.toplevel.fa.gz, and the genomic feature annotation GTF file was accessed at https://ftp.ensembl.org/pub/release-112/gtf/capra_hircus/Capra_hircus.ARS1.112.gtf.gz. The TEs of the *C. hircus* genome were annotated using the RepeatMasker (version 4.1.6) software (Tarailo-Graovac and Chen, 2009), with default blast mode *rmblastn* (version 2.14.0+). *De novo* TE annotations in goat were previously conducted by Dong et al. (2013), where they performed Repbase-dependent RepeatMasker annotation, together with RepeatModeller- and LTR_FINDER-based *de novo* repeat annotations. Their annotations were integrated into the Repbase-repeat libraries of current RepeatMasker version, we thus used “-species *C. hircus*” to call the priori annotations as the reference for our current genome assembly. To improve the coverage of TE annotation, the repeat library *FamDB* (CONS-Dfam_with RBRM_3.8) was also included for *rmblastn*. The RepeatMasker generated tables were then parsed to filter out repeats like rRNA, scRNA, snRNA, srpRNA, and tRNA. The makeTEgtf.pl script (http://labshare.cshl.edu/shares/mhammelllab/www-data/TEtranscripts/TE_GTF/makeTEgtf.pl.gz) was used to reformat RepeatMasker tables into GTF file for subsequent analysis. Each TE and ERVs in the table were given a unique identifier, with genomic location, element name, subfamily and class information extracted from the table and were included in the GTF file. Bedtools (-*intersect*) was used to define the genomic location of ERVs in intergenic, intronic, and exonic regions.

### 2.4 Transcriptomic identification of TEs and ERVs at subfamily and location levels

The quality of raw sequencing data was assessed using FastQC (https://www.bioinformatics.babraham.ac.uk/projects/fastqc/). Raw reads were trimmed and adapters were removed using trimmomatic-0.39-2 (Bolger et al., 2014). Trimmed reads were aligned to the *C. hircus* genome using STAR-2.7.4a (Dobin et al., 2013). Coding-gene and TE -derived reads were quantified using TEtranscripts (v2.2.3) and TElocal (v1.1.1) with their default parameters (Jin et al., 2015; Kabiljo et al., 2022). The aligned BAM file, together with two annotation GTF files for genes and TEs was used as the input data for TEtranscripts to identify TE subfamilies, while TElocal was used for single location TE and ERV identification. TE-derived transcripts were annotated according to the TE GTF file generated from the RepeatMasker. Because of the repetitive nature of TEs, there might be frequent multi-mapping and overlapping TEs, which may lead to bias to subsequent differential expression analysis. The following two steps were considered to minimize such impact. Firstly, RepeatMasker handles ambiguity of overlapping TEs or multi-mapping by scoring sequence features and context. Distal overlapping TEs were identified and reported separately in the output file, while overlapping TEs in proximal were fused. Then, TEtranscripts used equal weighting and expectation maximization strategies to avoid bias for differential expression analysis. If a read was mapped to multiple TEs, the read was equally weighted for each TE, to avoid bias by single mapping. The software use expectation maximization to estimate TE expression from multi-mapping reads, to ensure accurate quantification of TE expression. The strategy for such reads mapping and quantification are the same for all samples, resulting in comparable count matrix for subsequent differential expression analysis.

### 2.5 Differential expression analysis

**FIGURE 2:**
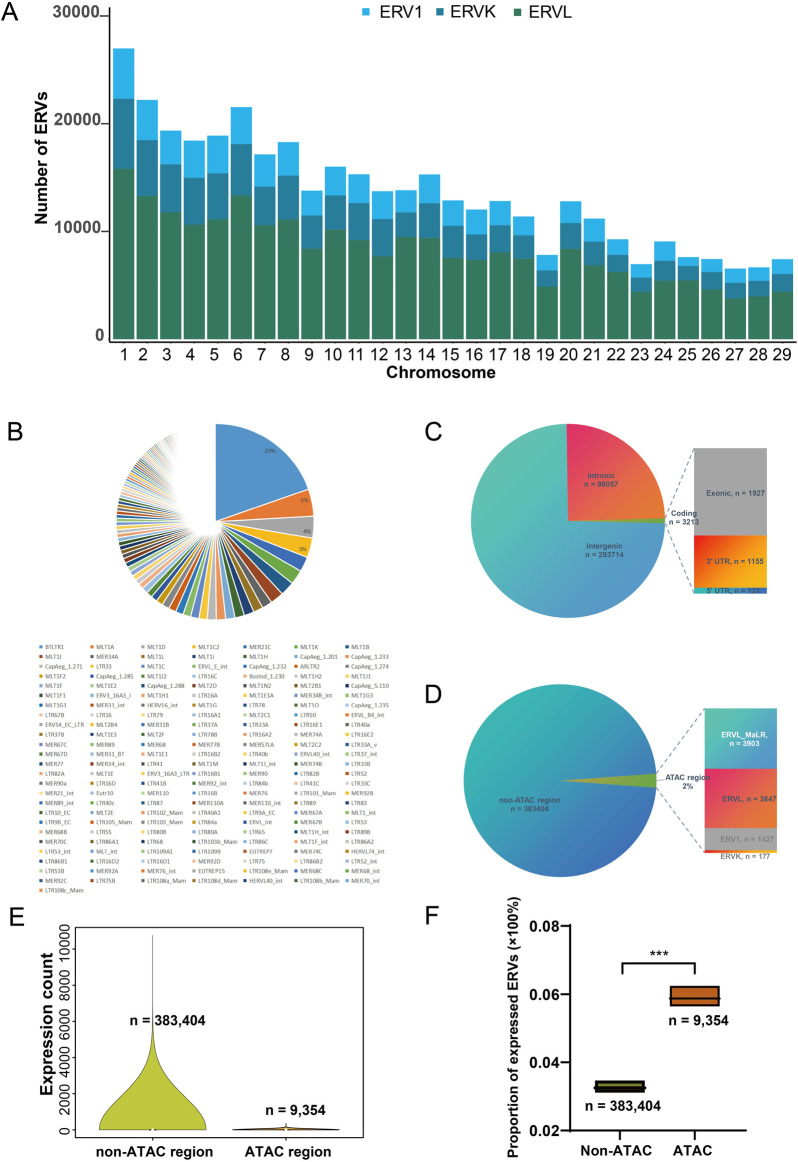
Genomic features of ERVs in the goat genome. **(A)** Number of ERVs in all goat chromosomes. **(B)** Pie chart representing the proportions of ERV families in the genome. **(C)** Distribution of ERV insertions in genomic contexts. UTR, untranslated region; Coding, regions from 5′UTR, exons to 3′UTR. Note that some TE elements were annotated to be both intronic and located in coding region due to alternative splicing, leading to double counting; and the number of non-redundant ERVs are 392,758. **(D)** Ratio of ERV insertions in open chromatin regions. ATAC region, those genomic fragments revealed to be open chromatin by transposase-accessible chromatin using sequencing (ATAC-seq) of liver and immune cells from goat (Foissac et al., 2019). **(E)** Distribution of expression levels in liver for ERVs located in open chromatin region. Shown expression levels were measured by total count of mapped reads. **(F)** Proportion of expressed ERVs (raw count > 0) within and outside of the ATAC-region (region with ATAC peaks) from normal goat liver and T cells (Foissac et al., 2019). Expression profile of liver from GSE93855 was used.

Following the generation of a count table for gene and TE transcripts, differential expression analysis of genes and TEs was performed using R package DESeq2 (v1.20.0) with default parameters (Love et al., 2014). Normalized count, defined as counts devided by sample-specific size factors, determined by median ratios method of normalization, was used for differential expression analysis, correlation analysis, and visualization. Differentially expressed TEs were identified at the subfamily level. Considering the huge number of TEs at the single location level, we focused on differentially expressed ERV reads, which are highly involved in various physiological and pathological conditions at the location level. Since most of the genomic ERVs have no single read mapped to the annotated region, ERVs with raw count >0 were arbitrarily defined as expressed and were subjected to subsequent differential expression analysis. A gene, TE, and ERV-derived transcript with false discovery rate (FDR-adjusted *P*-value) less than 0.005 was defined as significantly differentially expressed. The ERVs and nearby genes at the location level were visualized using the Integrative Genomics Viewer (IGV) (version 2.18.2) with the read alignments (BAM) file. Since there is possibility that the proposed expression of candidate TE-derived transcript might be the by-product of the host gene expression, those TEs annotated to be located in coding regions (exonic, 5′UTR, and 3′ UTR in Figure 2C) were excluded in the differential expression analysis. Volcano plots showing differential expression of TEs and ERVs were made using the R package *ggplot2* (version 3.5.1). Heatmaps were created using the R package *pheatmap* (version 1.0.12). Correlations between ERV expression level and coding gene levels were measured by Spearman’s rho correlation.

## 3 Results

### 3.1 Genomic and transcriptomic features of TEs

Distribution of classes, families, and subfamilies of TEs in the *C. hircus* genome was systematically annotated. Their expression pattern in multiple tissues under different physiological and pathological conditions was investigated at TE subfamily and location levels (Figure 1A). In the genomic context, 49.1% of the goat genome is composed by TE sequences, with 25.98% are LINEs, 10.24% SINEs, 3.98% LTRs (ERVs) and 1.97% are DNA transposons (Figure 1B; Supplementary Table S1). These TE sequences could be divided into about 749 (average number of the TE subfamilies in transcriptome of analyzed tissues, range from 622 to 794) subfamilies, of which the majority are DNA transposons and LTR transposons in the transcriptome (Figure 1C; Supplementary Table S2).

Though they account for near half the genome sequence, TE-derived reads only account around 10% of the transcriptome in various tissues (Figure 1D). Consistent with the genomic sequence ratio (Figure 1B), abundance of SINEs and LINEs in the transcriptome is the highest among the ∼700 TE subfamilies (Figure 1E; Supplementary Table S2). And the SINEs and LINEs derived transcripts showed a high abundance in the transcriptome of blastocyst and endometrium, partially due to a high level of transcriptomic variations at both subfamily (Supplementary Figure S1) and location (Supplementary Figure S2) levels within the group. In spite of the sample heterogeneity, SINEs and LINEs derived transcripts each account for around 5% on average, while DNA TEs and LTR TEs each account for 1%, of the transcriptome across the tissues (Figures 1D, E). The comparable expression abundance of each TE family in different tissues and cells suggested constitutive expression of TEs in the transcriptome. Indeed, the constitutive expression of TEs is robust in physiological conditions like skins sampled from the normal and hyperpigmented goats, since few dysregulated TEs were observed in such condition (Supplementary Table S3, GSE69812). Notably, in pathological conditions, such as tissues infected with the Peste-des-petits-ruminants virus (PPRV), a large portion of the TE subfamilies are dysregulated with a stringent cutoff (FDR-adjusted *P*-value <0.005) for significantly differential expression: 165 (21.5%) in lung, 155 (20.2%) in spleen, and 654 (85.3%) in caecum were altered under PPRV challenge (Supplementary Table S3, GSE130552). This observation indicated extensive dysregulation and active involvement of TE expression in pathological conditions like external infection.

In addition to pathological challenge, regulation of TEs expression might also be variable across tissues (Figure 1F). In the 27 tissue-specific TEs (Figure 1F), half of them were LTR (which defines ERV) derived reads, indicating a variable nature of ERVs among the TE transcripts. Moreover, many ERVs have the potential to code for proteins, and are more active than other types of TEs in various processes. We thus focused on ERVs in subsequent analyses.

### 3.2 Genomic and transcriptomic features of ERVs

There are three major families of ERVs in the goat genome, ERV1, ERVK, and ERVL. These ERVs are located in all goat chromosomes, with the number of ERVs increasing along with chromosomal length (Figure 2A). Among 176 ERV subfamilies, the most abundant ERVs are BTLTR1, MLT1A, MLT1D, and MLT1C2 at the location level (Figure 2B). In the 392,758 non-redundant ERV insertions, most are located in intergenic regions, and 0.8% (n = 3213 ERV locations/insertions) of them located in coding regions (Figure 2C). We further investigated whether these ERVs are located in heterochromatin region or accessible chromatin region. The ATAC-seq data from normal goat liver and T cells, a reference of chromatin accessibility in ruminants (Foissac et al., 2019), was used to assess accessible chromatin regions. Based on the released open chromatin positions in the ATAC-seq data, we found that 2% (n = 9,354 ERV locations/insertions) of the genomic ERVs located in open chromatin region in goat liver or immune cells (Figure 2D). Those ERVs located outside of the open chromatin region show more various expression in goat tissues, due to the large number of such ERVs (Figure 2E). Since most of the genomic ERVs have no mapped read (raw count = 0) in the transcriptome, we thus measured the proportion of expressed ERVs (raw count >0) within and without the ATAC-region (region with ATAC peaks). The proportion of expressed ERVs located in the ATAC-region was significantly higher than that of non-ATAC regions (0.058% vs 0.033%, *P* < 0.001, Figure 2F). However, more than 90% of the 392,758 ERVs remain silenced in analyzed tissues under physiological conditions (Figure 2F), consistent to the well-established knowledge that most ERVs are silenced in the genome. We then moved on to investigate where and how ERVs may become dysregulated in goat tissues.

### 3.3 Dysregulated ERVs in the reproduction system

**FIGURE 3:**
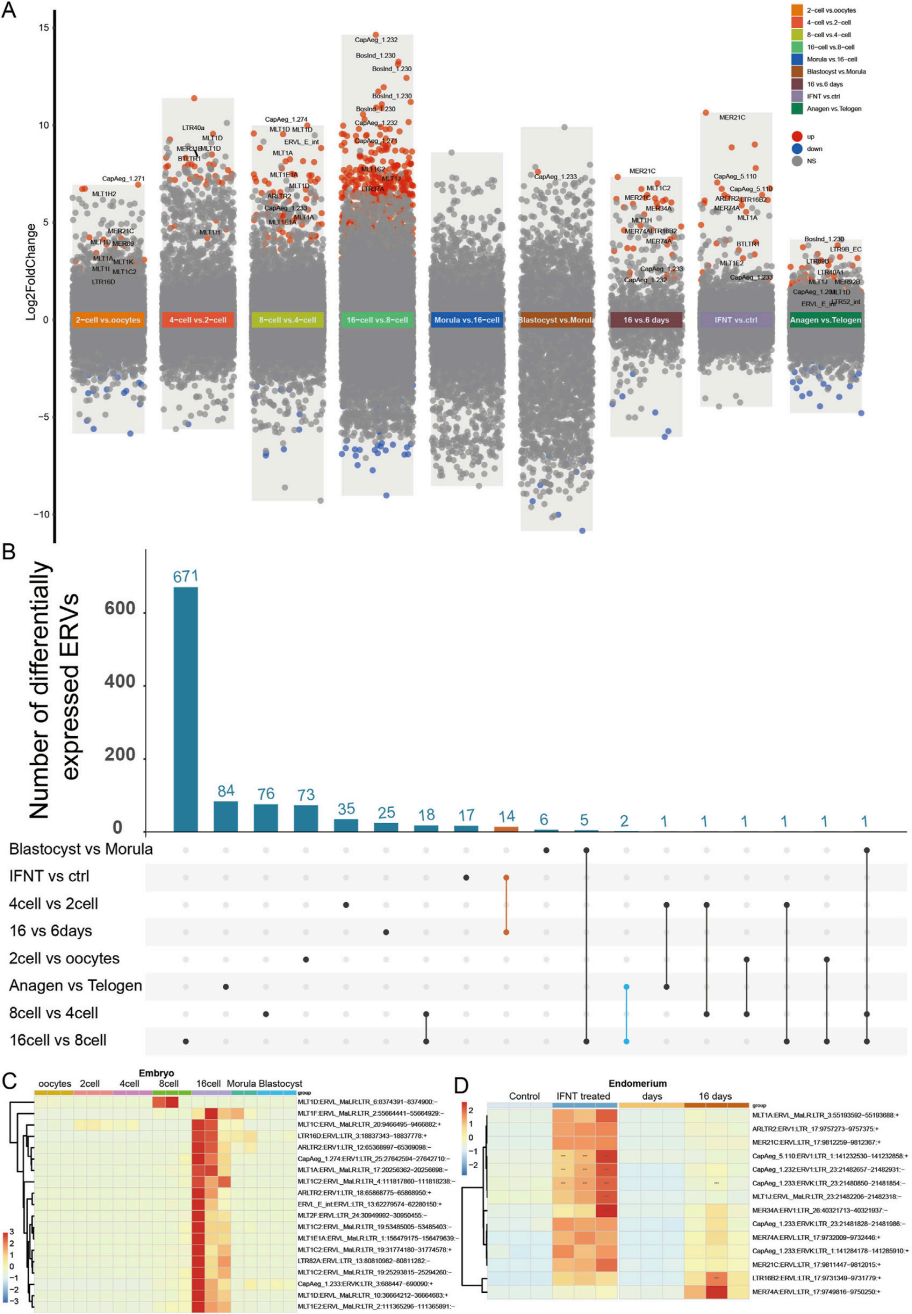
ERVs activated in embryo and peri-implantation endometrial epithelium. **(A)** Differentially expressed ERVs in embryos, endometrium, and skin at different stages. Different background colors were used to show different comparisons as marked by the text. Red dots, significantly upregulated ERVs; blue dots, significantly downregulated ERVs; grey dots, non-significant ERVs; vs, *versus*. ERVs with false discovery rate (FDR-adjusted P-value) < 0.005 were defined as significantly differentially expressed. “16 vs. 6 days”, ERVs from endometrial epithelium at 16 days peri-implantation compared to 6 days; “INFT vs ctrl”, INFT treated endometrial epithelium compared to control group; “Anagen vs Telogen”, ERVs from skin biopsies sampled at active growth phase (anagen) compared to resting phase (telogen). For detailed differential expression, please refer to Supplementary Table S4. **(B)** Number of activated ERVs shared by different comparisons. Orange line, activated ERVs shared by different endometrium (listed in Supplementary Table S5); blue line, activated ERVs shared by endometrial epithelium and embryos. **(C)** Heatmap of typical ERVs altered in 16-cell and 8-cell embryos. **(D)** Heatmap of dysregulated ERVs shared by different endometrium [N = 14, orange line in **(B)**]. log2 transformed counts were used for expression quantification. ERVs were clustered according to expression pattern.

Consistent with the robustness of TEs expression in physiological conditions, no ERVs were significantly altered during skin pigmentation (Supplementary Table S4, GSE69812). Since ERVs play essential roles during embryo development, we investigated whether any ERVs are differentially expressed in goat embryos under different developmental stages. Differentially expressed ERVs were also explored in the ovary and the endometrium (Supplementary Table S4), where the expression regulation might be cooperated with the embryo to ensure success reproduction. We found that among the 12,711 expressed ERVs, most dysregulated ERVs are activated during embryo development, and reached peak at 16-cell embryo (Figures 3A–C). In particular, there were 642 ERVs upsregulated (log_2_Fold Change [logFC] > 0, FDR-adjusted *P*-value [*P*_adj_] < 0.005) and 57 ERVs downregulated (logFC < 0, *P*_adj_ < 0.005) in 16-cell embryo compared with 8-cell embryo (Figures 3A, B). For instance, BosInd_1.230:ERV1:LTR (chr1:12676110-12676292, logFC = 13.265, *P*_adj_ = 4.867 × 10^−22^), CapAeg_1.232:ERV1:LTR (chr1:143876497-143876779, logFC = 14.649, *P*_adj_ = 1.135 × 10^−20^), and BosInd_1.230:ERV1:LTR (chr18:60592269-60592640, logFC = 11.199, *P*_adj_ = 1.848 × 10^−18^) are the most highly activated ERVs in 16-cell embryos (Figure 3A; Supplementary Table S4, GSE129742), when the genome of the zygote begins to transcribe its own genes. During this stage, the genome is of high accessibility, rendering the extensive expression of ERVs as well. In the late embryo stage, the blastocyst, only one ERV CapAeg_1.233:ERVK:LTR (chr1:6646491-6648140, logFC = 7.603, *P*_adj_ = 0.0022) was upregulated compared with morula.

In peri-implantation endometrial epithelium, there were only 1,024 ERVs expressed, among which there were 32 upregulated and 6 downregulated ERVs at 16 days post-implantation compared to 6 days endometrial epithelium (Figure 3A; Supplementary Table S4, GSE108557). None of these ERVs are overlapped with those dysregulated in embryos (Figure 3B), partially due to limited number of analyzed ERVs in endometrium. Since IFNT signaling is essential for implantation, there were a dozen ERVs upregulated in endometrial epithelial cells with IFNT treatment, which were consistent in 16 days post-implantation endometrial epithelium compared to 6 days (Figures 3C, D; Supplementary Table S4, GSE184110). In addition to endometrium, some ERVs (BosInd_1.230:ERV1 and LTR89B:ERVL) were also activated in skin during rapid growth of the hair follicles (Figures 3A, B), indicating the importance of cell proliferation state for ERV expression.

### 3.4 Dysregulated ERVs in response to infections

**FIGURE 4:**
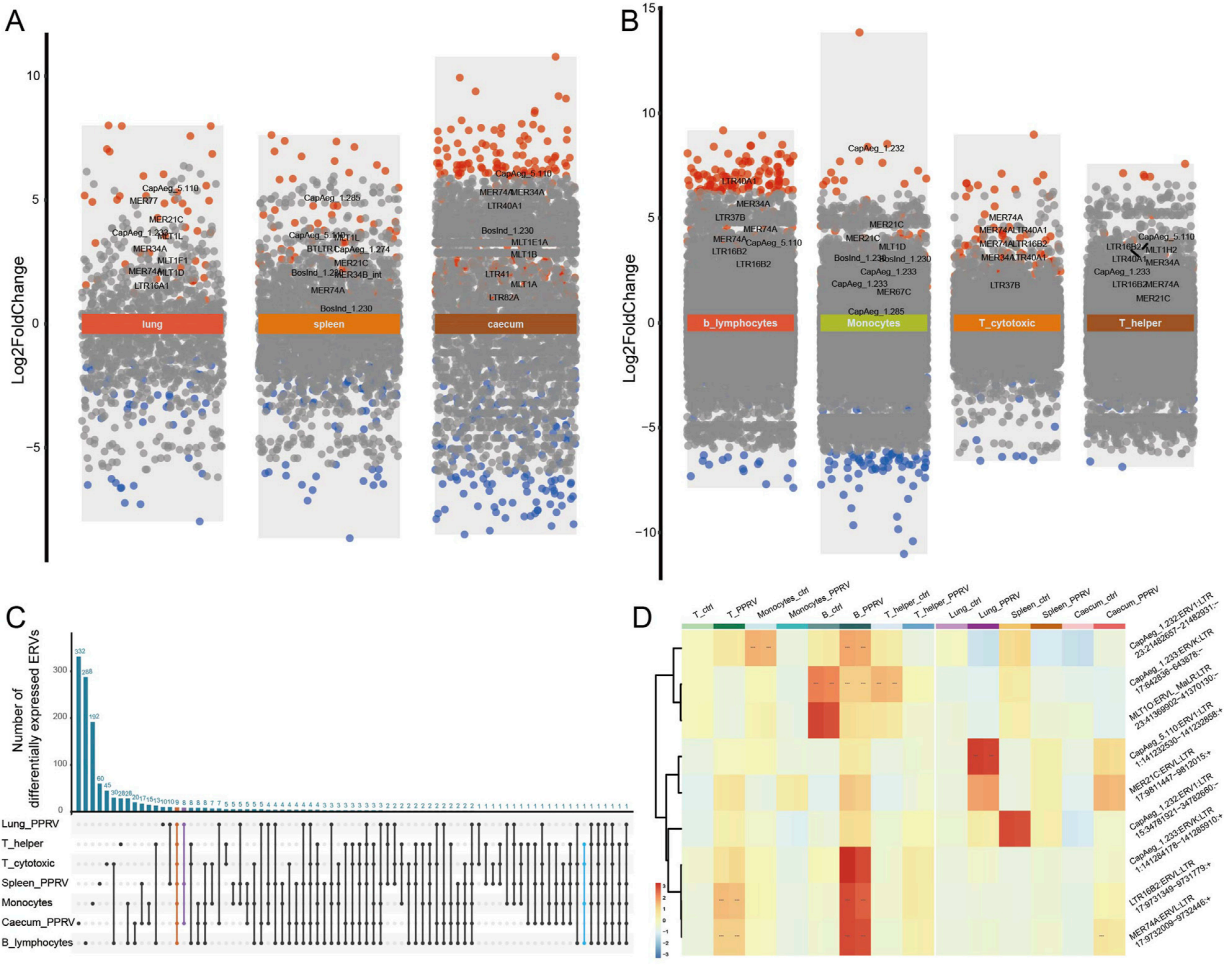
ERVs activated in tissues and immune cells under infection. **(A)** Differentially expressed ERVs in PPRV infected lung, spleen, and caecum. Different background colors were used to show different comparisons as marked by the text. Red dots, significantly upregulated ERVs; blue dots, significantly downregulated ERVs; grey dots, non-significant ERVs. ERVs with false discovery rate (FDR-adjusted P-value) < 0.005 were defined as significantly differentially expressed. For detailed differential expression, please refer to Supplementary Table S4. **(B)** Differentially expressed ERVs in PPRV infected monocytes and lymphocytes. **(C)** Shared ERVs across different tissues or cells under PPRV infection. **(D)** Heatmap of typical ERVs in response to infection. log2 transformed counts were used for expression quantification. ERVs were clustered according to expression pattern.

In addition to the cell proliferation-responsive ERVs, we recognized numerous activated ERVs in response to virus infection in the lung, spleen, caecum, and immune cells (Figures 4A, B; Supplementary Table S4). In goats infected by the Peste-des-petits-ruminants virus (PPRV, a single strand RNA virus), there were more upregulated ERVs in caecum and B cells (Figures 4A, B). 327 out of 4,542 ERVs were upregulated and 164 downregulated in caecum under PPRV infection, with 20 ERVs consistently upregulated in all three tissues (Supplementary Table S4). The most significantly upregulated were MER74A:ERVL:LTR (chr17:9732009-9732446), CapAeg_5.110:ERV1:LTR (chr1:141232530-141232858), and MER34A:ERV1:LTR (chr26:4,0321713-4,0321937). Intriguingly, MLT1E1A:ERVL_MaLR:LTR (chr11:29358543-29359075) was significantly upregulated in caecum (logFC = 3.012, *P*_adj_ < 1.0 × 10^−114^) but downregulated in lung (logFC = −1.673, *P*_adj_ = 5.222 × 10^−11^). Nine ERVs were consistently dysregulated in the PPRV-infected tissues and immune cells (Figures 4C, D), for instance, CapAeg_5.110:ERV1 and MER21C:ERVL were upregulated in all tissues and immune cells. Notably, we observed opposite direction of ERV activation between monocyte and lymphocytes, namely, those ERVs upregulated in PPRV-infected lymphocytes and tissues were downregulated in PPRV-infected monocyte (Figures 4C, D). This observation was in accordance with different anti-virus roles of lymphocytes and monocytes.

Intriguingly, DNA virus, like Orf virus (ORFV), and non-viral infection (e.g., *Mycoplasma agalactiae* and *M. avium subsp. Paratuberculosis*) activate less ERVs in the transcriptome (Supplementary Table S4, GSE121725, GSE30379, and GSE117799), indicating that those RNA-virus-derived endogenous ERVs may be hitchhiking on the infection processes of external RNA virus infection.

### 3.5 Regulatory mechanism of dysregulated ERVs by proximal genes

**FIGURE 5:**
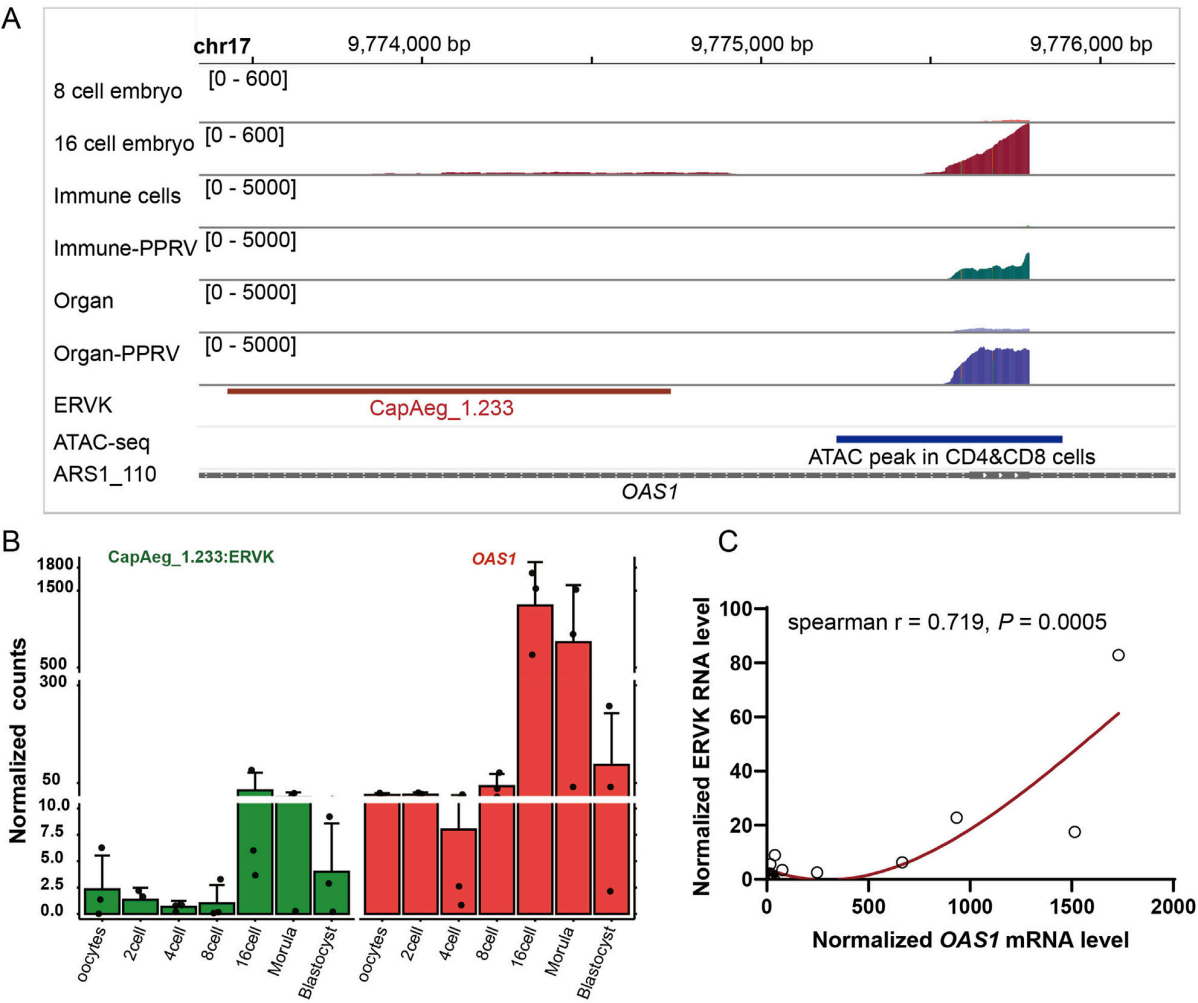
Dysregulated CapAeg_1.233:ERVK and *OAS1* in embryo. **(A)** Expression and location of CapAeg_1.233:ERVK and *OAS1* in chromosome 17 in embryo and infected organs and cells. Shown peaks were measured by summed count of mapped reads in all samples within the group. ATAC-seq, quantified ATAC-seq peaks from goat T cells generated by the Functional Annotation of Animal Genomes (FAANG) project (Foissac et al., 2019). **(B, C)** Expression and correlation of CapAeg_1.233:ERVK and *OAS1* in embryo. Expression shown in normalized count, using median of ratios method of normalization in Deseq2. Different colors were used to show different groups.

We then investigated how and why those ERVs are dysregulated under certain conditions. ERVs altered in various conditions (indicated by Figures 3C, 4C) were subjected to subsequent analysis (Supplementary Table S5). Among the commonly altered 35 ERVs, five out of the 14 differentially expressed ERVs shared by 16 days-post-implantation endometrial epithelium and IFNT treated endometrial epithelial cells located around an important interferon-induced gene, *OAS1*. And in nine differentially expressed ERVs in PPRV-infected tissues and cells, there were three located within *OAS1*. Though *OAS1* activated in both embryo development and virus infection, the proximally activated ERVs are different, indicating specific physiological functions of distinct ERVs. LTR16B2:ERVL and MER74A:ERVL were significantly upregulated under infection (Supplementary Figures S3A, B), and showed positive correlation with the expression of *OAS1* (Supplementary Figures S3B, C), whereas CapAeg_1.233:ERVK was specifically upregulated in embryos (Figure 5A) and also showed positive correlation with *OAS1* (Figures 5B, C). Notably, these ERVs are located in the intronic region of *OAS1*, rendering the possibility that the proposed expression of candidate TE-transcript might be the by-product of the host gene expression. Indeed, LTR16B2 and MER74A are such cases, since the reads distributed equally within and outside of the TE along the intron (Supplementary Figure S3A). And CapAeg_1.233:ERVK was reasonably expressed independently, with peaks exactly in TE region and no reads outside of TEs were observed along the intron (Figure 5A). We thus conclude that though ERVs around OAS1 were proposed to be frequently altered in various conditions, only CapAeg_1.233:ERVK showed robust and specific expression during embryo development. Whether infection may induce the expression of MER74A and LTR16B2, independent of infection-induced *OAS1*, remain to be determined. Because of the incomplete of the annotation of goat genome, we cannot rule out whether there are an unknown infection-inducible *OAS1* isoform or ERV-derived lncRNA.

**FIGURE 6:**
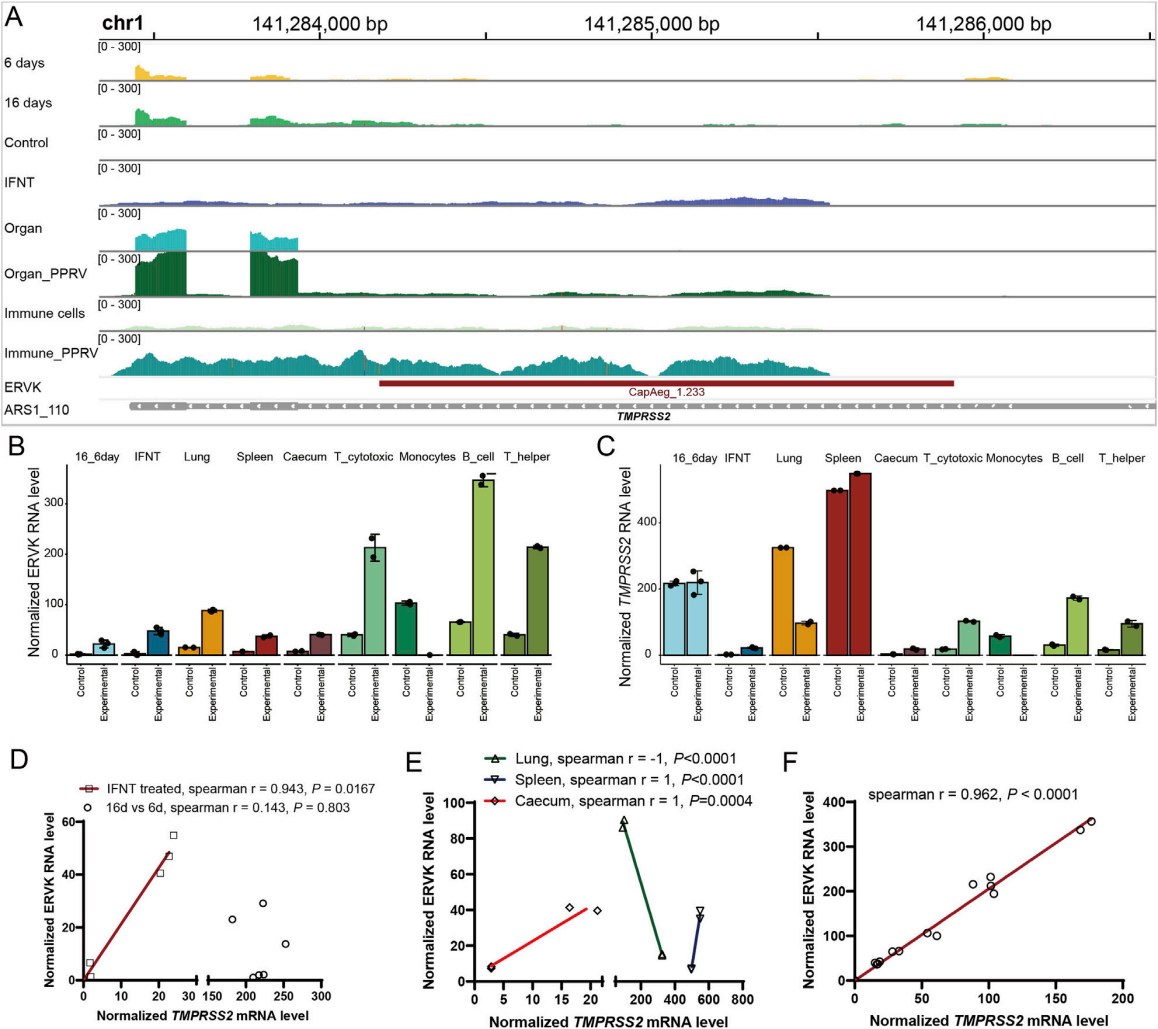
Correlation of CapAeg_1.233:ERVK and *TMPRSS2* expression in response to infection. **(A)** Expression and location of CapAeg_1.233:ERVK and *TMPRSS2* in chromosome 1 during endometrium development and infection challenges. **(B, C)** Expression of CapAeg_1.233:ERVK **(B)** and *TMPRSS2* **(C)** in chromosome 1 in various tissues. **(D)** Correlation between of CapAeg_1.233:ERVK and *TMPRSS2* during endometrium development. **(E, F)** Correlation between of CapAeg_1.233:ERVK and *TMPRSS2* expression in response to PPRV infection. Expression shown in normalized count, using median of ratios method of normalization in Deseq2. Simple linear regression was used to measure the co-expression between ERVs and their proximal genes.

In addition to the *OAS1* region, CapAeg_1.233:ERVK:LTR (chr1:141284178-141285910) proximal to another important immune gene, *TMPRSS2*, was altered during endometrium development and under infection (Supplementary Table S5; Figure 6). It shows positive correlation with the expression of *TMPRSS2* in IFNT-treated endometrium (Figure 6D), PPRV-infected caecum, spleen (Figure 6E), and immune cells (Figure 6F). Strangely, *TMPRSS2* was downregulated in PPRV-infected lung (Figure 6C), leading to unexplainable negative correlation between CapAeg_1.233:ERVK:LTR and *TMPRSS2* in lung (Figure 6E). Some other infection-responsive genes like *IFI144*, *MX1, MX2, IFIT3,* and *HLA-DOA* were also observed proximal to those altered ERVs (Supplementary Table S5), and showed positive co-expressions with their proximal ERVs, wherever they located (Supplementary Figure S4). We therefore propose that dysregulated proximal genes, which are active in response to the respective conditions, contribute to the dysregulation of nearby ERVs.

## 4 Discussion

TE-derived transcripts account for a non-negligible proportion of a mammalian transcriptome (Bourque et al., 2018; Dopkins and Nixon, 2024). However, most standard expression analyses ignored such reads due to the lack of tools that allow easy inclusion of TE-derived reads (Lanciano and Cristofari, 2020). In goat, the transcriptomic feature of TEs are missed, limiting the full understanding of the goat genome. In this study, we took advantage of TEtranscripts and TElocal in analyses of series of transcriptomes of goat tissues. Since TEtranscripts is highly dependent on the quality of the genomic annotation, and this is problematic for less studied species like the goat, we manually curated a GTF file for TE annotation in goat. Then we recognized the abundance of TE-derived reads in transcriptomes of goat tissues and investigated the expression pattern of ERV-derived transcripts in various tissues and conditions. We found that TEs are constitutively expressed in the transcriptome of tissues and cells, accounting for 10% of the transcriptome. ERVs are actively altered in some conditions, especially during embryo development and in response to infection (Supplementary Tables S3, S4). Specially, we showed that ERVs on chromosome 17 respond to different physiological and pathological conditions, and are co-expressed with their proximal coding genes. These results may benefit goat-based genomic and transcriptomic research.

TEs in the goat genome have been investigated when the reference genome was released (Dong et al., 2013; Dong et al., 2015; Belay et al., 2024). The ratio of TEs, especially LTRs, is consistent with our re-analyses using another genome assembly, supporting the reliability of the TE annotation. However, the detailed genomic features of TEs and ERVs were unclear in the goat (Chang et al., 2024), while they are well-studied in sheep and other farm animals (Klymiuk et al., 2003; Baillie et al., 2004; Arnaud et al., 2008; Garcia-Etxebarria et al., 2014). It is therefore worthwhile to conduct a deep annotation of TEs at the family and location levels for the goat genome. We herein curated the first GTF file for TE annotation in goat, which is publicly available for further validation and use.

Being an essential part of the genome sequences, TEs play multiple roles in the evolution, structure and function of mammal genome (Lanciano and Cristofari, 2020; Horvath et al., 2017; Bourque et al., 2018), such as in expression regulation (Lanciano and Cristofari, 2020; Ivancevic et al., 2024; Yu et al., 2023; Branco and Frost, 2023). Moreover, the abundance of TEs is constitutive in the transcriptome, though most TE sequences are silenced. Among those expressed TEs, ERVs gained most attention, since ERV-derived genes drive the evolution of placental mammals (Chuong, 2018; Mi et al., 2000; Bourque et al., 2018; Haig, 2012), and even function in other non-mammals like birds (Chen et al., 2022). Indeed, we found that in goat embryo, a thousand of ERVs are dysregulated during embryo development, especially in the 16-cell stage, when the zygote’s genome is activated (Deng et al., 2019). During embryo development, the cell proliferates quickly, and the chromatins are highly open, rendering the concomitant expression of ERVs. Similarly, the transcription of the host cell becomes active when challenged by infection, ERVs are also highly activated. Notably, the interferon-induced gene *OAS1*, upregulated in both development and infection, resulting in the activation of its nearby ERVs. Yet it is unclear why *OAS1* is upregulated in embryo development and what’s the impact of ERV activation in these progresses. In particular, these endogenous retroviruses become active when there are exogenous retroviruses, or RNA virus (PPRV in this study) infections, while DNA virus or bacterial infection induce few ERV expression. Surely there are interactions between the contemporary retroviruses and their endogenized ancestors (Kyriakou and Magiorkinis, 2023). This is supported by a recent human study showing the activity of certain ERVs in the colon of HIV reservoirs (Dopkins et al., 2024a). These observations suggested that active transcription event of the host might be hijacked by ERVs (Asimi et al., 2022; Grow, 2022), which may lead to subsequent impact on the host cells (Dopkins and Nixon, 2024; Ivancevic et al., 2024; Wang et al., 2024; Yu et al., 2024; Guo et al., 2024; Dopkins et al., 2024b; da Silva et al., 2024). Notably, such TEs should be interpreted with caution, since there is possibility that the proposed expression of candidate TE-transcript might be the by-product of the host gene expression. This bias should be particularly checked when the TEs were in exonic and intronic regions. Moreover, there might be overlapping TEs due to their repetitive nature. Though we did not conduct experimental validation since the study covers various types of tissues and conditions, we resolved ambiguity using a series of strategies. Further experimental validations are undoubtedly warranted for the above issues.

In summary, in this study we curated a GTF file for TE annotation and generated the first TE-derived transcriptomes across goat tissues. The expression pattern of ERV-derived transcripts in various tissues and conditions was comprehensively explored. These results may benefit goat-based genomic and transcriptomic researches. It may also enhance the understanding and treatment of infection threats for goat farming. The annotation of TEs might be biased, since the reference genome keeps updating due to the rapid development of sequencing techniques. Annotating these structural variations and repetitive elements using more recent genome assemblies, or using full-length sequencing data, followed by experimental validations, will undoubtedly improve future TE-related research.

## Data Availability

The data presented in the study are available in the GEO repository, accession numbers listed in Table 1. All data generated or analyzed during this study are included in this published article and its Supplementary Material. Codes and GTF files for TE annotation at the family and location levels are publicly accessible through Figshare at https://figshare.com/articles/dataset/GTF_files_for_annotating_transposable_elements_in_i_Capra_hircus_i_goat_genome/27898515 (DOI: 10.6084/m9.figshare.27898515). These files can also be accessed through the following links: https://drive.google.com/file/d/12CllX4cFKJ5us8aq0I-xDVFDydyaRTpc/view?usp=drive_link for TE family annotation, and https://drive.google.com/file/d/1NpE_5eZOOAdcsUaJuks446YzqnaBjk2a/view?usp=drive_link for TE location annotation.
