# Supplementary material for: Multi-tissue transcriptomic characterization of endogenous retrovirus-derived transcripts in Capra hircus
Source: Front Genet. 2025 Mar 19;16:1544330. doi: 10.3389/fgene.2025.1544330 (PMC11962033; doi:10.3389/fgene.2025.1544330)
Supplement: Supplementary file 3 [file DataSheet1.pdf]

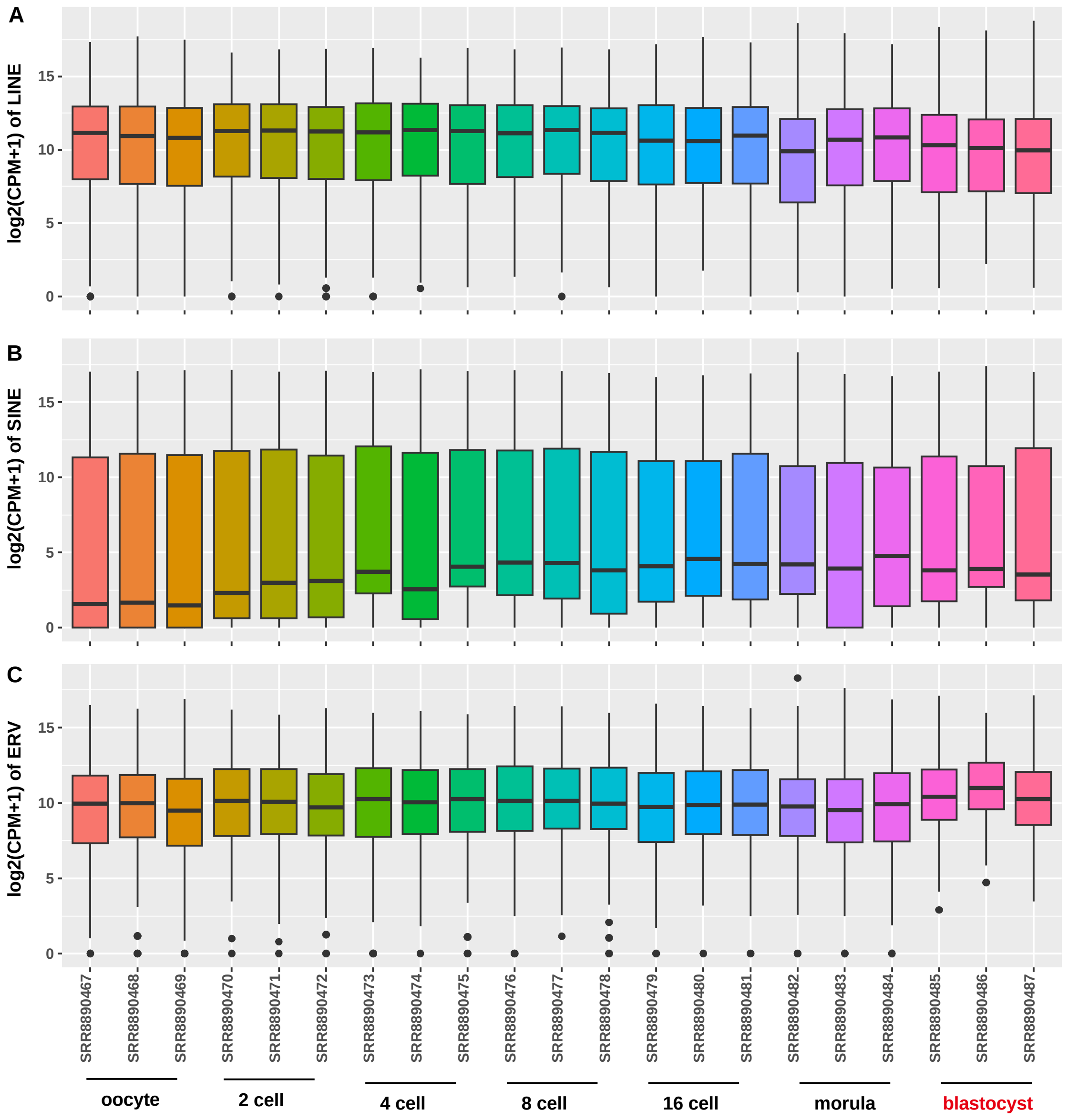

Figure S1. Distribution of TE family derived count in transcriptome of the embryo samples. A, distribution of LINE-derived count at subfamily level. B, distribution of SINE-derived count at subfamily level. C, distribution of ERV-derived count at subfamily level. Shown expression measured by log2 transformed count per million reads (CPM). Data sourced from GSE129742, samples at the blastocyst stage were marked in red, to show variations within this group.

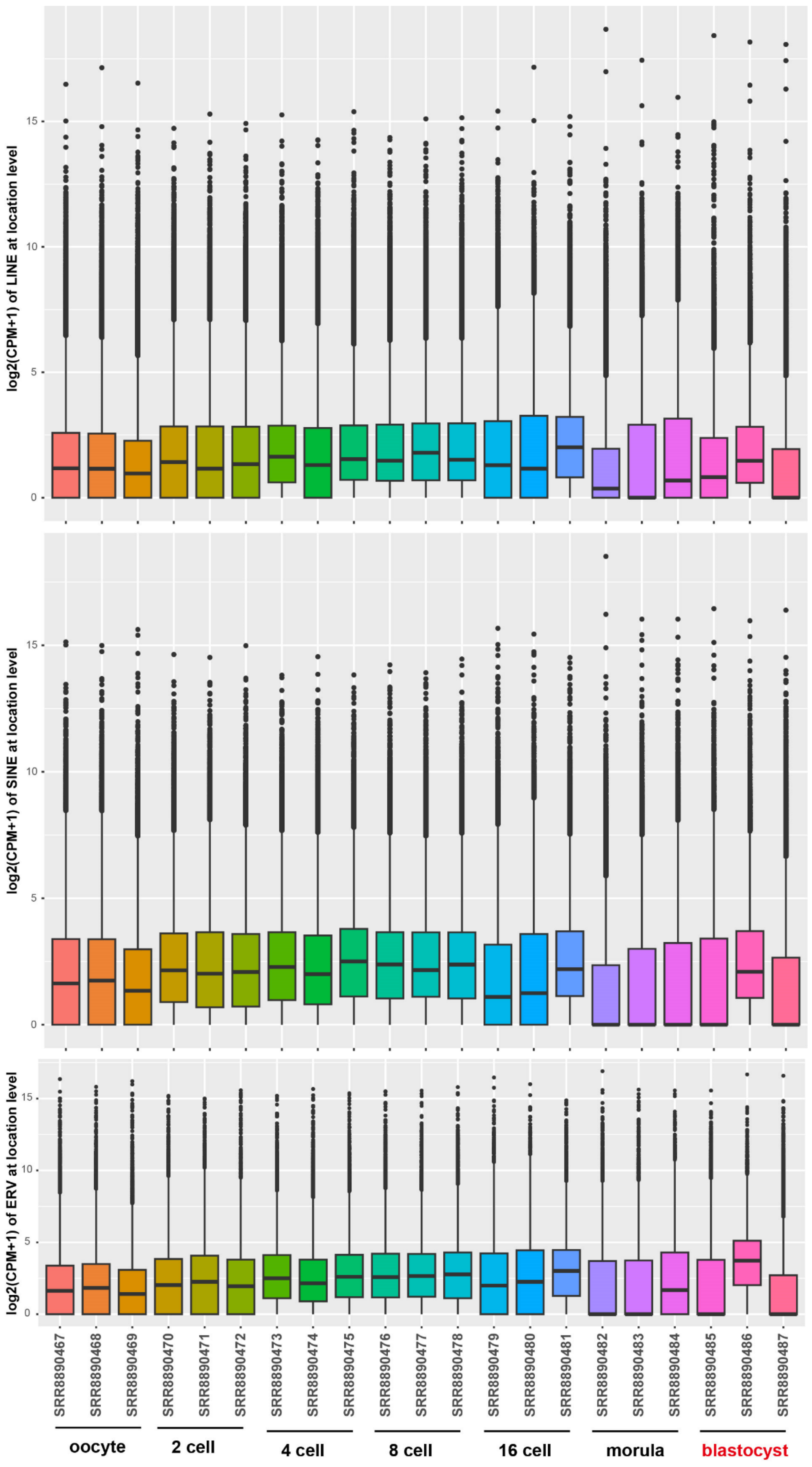

log2(CPM+1) of LINE at location level
log2(CPM+1) of SINE at location level
log2(CPM+1) of ERV at location level
0
5
10
15
SRR8890467
SRR8890468
SRR8890469
SRR8890470
SRR8890471
SRR8890472
SRR8890473
SRR8890474
SRR8890475
SRR8890476
SRR8890477
SRR8890478
SRR8890479
SRR8890480
SRR8890481
SRR8890482
SRR8890483
SRR8890484
SRR8890485
SRR8890486
SRR8890487
oocyte
2 cell
4 cell
8 cell
16 cell
morula
blastocyst

Figure S2. Distribution of single TE location derived count in transcriptome of the embryo samples. A, distribution of LINE-derived count at location level. B, distribution of SINE-derived count at location level. C, distribution of ERV-derived count at location level. Shown expression measured by log2 transformed count per million reads (CPM). Data sourced from GSE129742, samples at the blastocyst stage were marked in red, to show variations within this group.

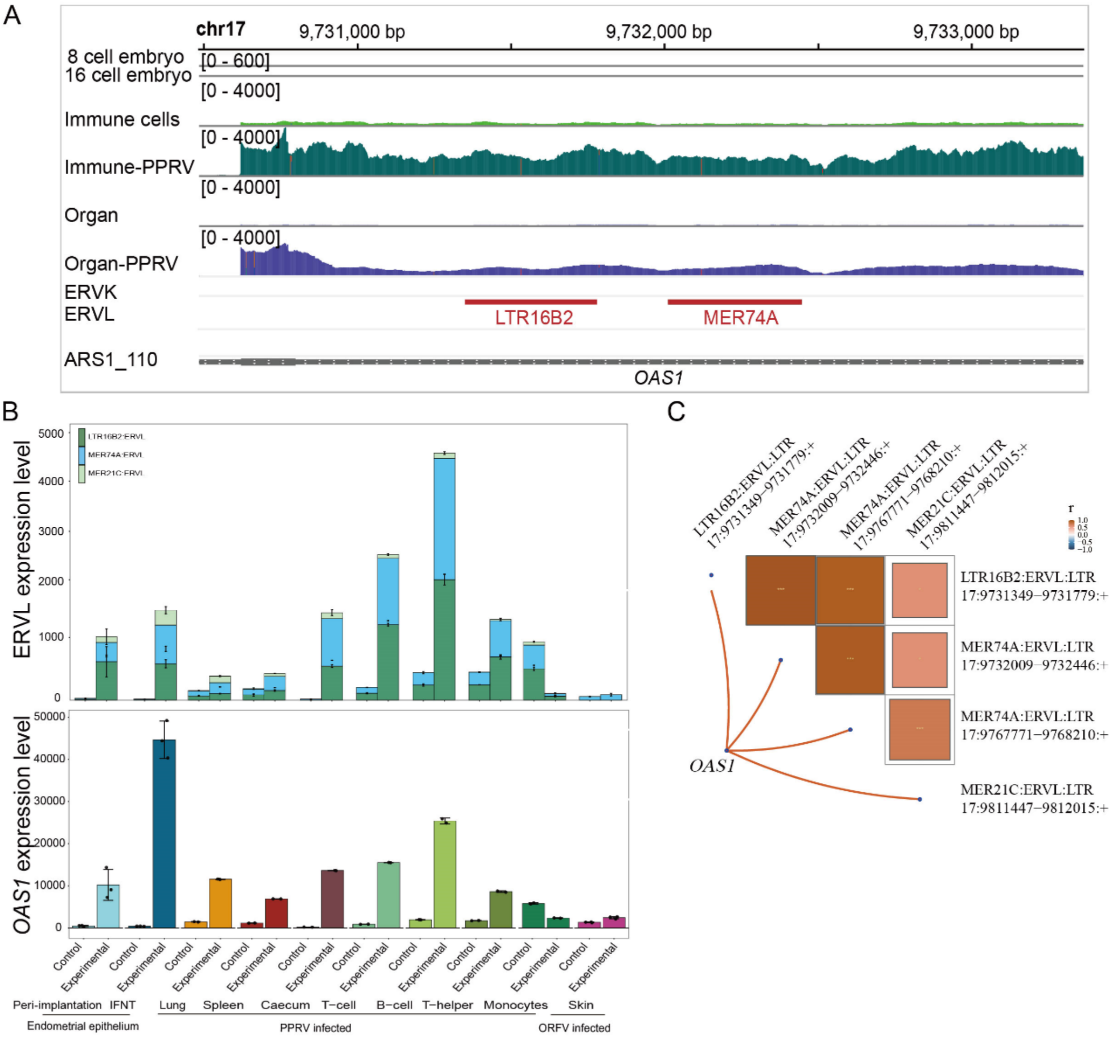

Figure S3. Dysregulated ERV around *OAS1* in response to PPRV infection. A, expression and location of ERVs and *OAS1* in chromosome 17 in embryo and infected organs and cells. Shown peaks were measured by summed count of mapped reads in all samples within the group. B, expression of ERV and *OAS1* in endometrium and infected tissues. C, correlation between the expression level of three ERVs and *OAS1* in endometrium and infected tissues. Expression shown in normalized count, using median of ratios method of normalization in Deseq2. Different colors were used to show different groups.

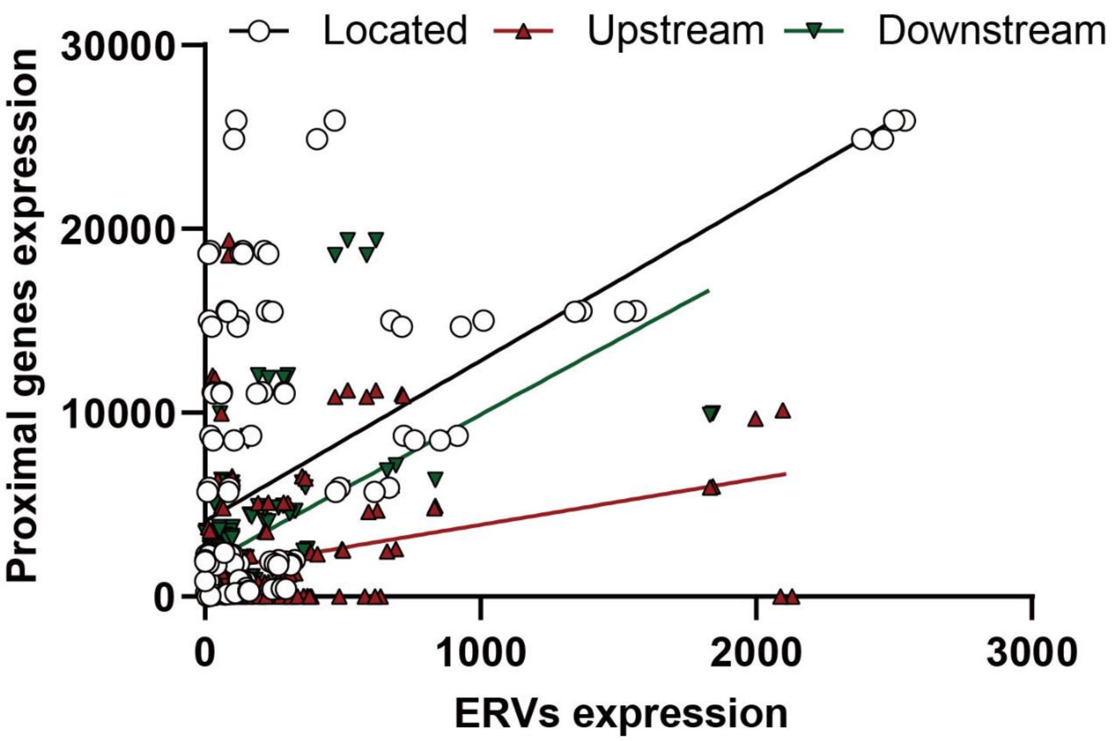

Figure S4. Correlation between the expression level of ERVs and their proximal genes in PPRV-infected tissues and cells. Counts of ERVs and genes listed in Table S5 were retrieved from the respective datasets (GSE130552 and GSE132429). Simple linear regression was used to measure the co-expression between ERVs and their proximal genes. Located, genes where the ERVs are located within their intronic region, Goodness of fit $R^2 = 0.3114$, *P-value* < 0.0001; Upstream, nearest gene upstream of the target ERV, Goodness of fit $R^2 = 0.06821$, *P-value* < 0.0001; Downstream, nearest gene downstream of the target ERV, Goodness of fit $R^2 = 0.278$, *P-value* < 0.0001.
